# DERMATOLOGICAL MANIFESTATIONS IN HIV-INFECTED PATIENTS AT A TERTIARY CARE HOSPITAL IN A TRIBAL (BASTAR) REGION OF CHHATTISGARH, INDIA

**DOI:** 10.4103/0019-5154.57609

**Published:** 2009

**Authors:** Harminder Singh, Prabhakar Singh, Pavan Tiwari, Vivek Dey, Navin Dulhani, Amita Singh

**Affiliations:** *From the Departments of Pharmacology, Government Medical College, Jagdalpur (CG), India.*; 1 *From the Departments of Anatomy, Government Medical College, Jagdalpur (CG), India.*; 2 *From the Departments of Dermatology, Government Medical College, Jagdalpur (CG), India.*; 3 *From the Departments of Medicine, Government Medical College, Jagdalpur (CG), India.*; 4 *From the Departments of Physiology, Government Medical College, Jagdalpur (CG), India.*

**Keywords:** *Human immunodeficiency virus*, *National Aids Control Organisation*, *people living with HIV/AIDS*

## Abstract

**Background:**

Cutaneous disorders during HIV infection are numerous and skin is often the first and only organ affected during most of the course of HIV disease. Some Cutaneous disorders reflect the progression of HIV disease; though the relation is still controversial.

**Aims:**

The objective of this study, conducted at a tertiary care centre in Bastar, Jagdalpur, is to estimate the status of cutaneous manifestation in HIV-infected patients and its relationship with CD4 cell counts.

**Methods:**

We enrolled 137 HIV positive subjects. Demographic information such as age, gender, weight, height, socioeconomic status, and educational status were recorded. Laboratory parameter (CD4 counts) and treatment regimen were noted. Patients were examined for skin disorders by a dermatologist. Data were analyzed using chi-square test for categorical variables.

**Results:**

Majority of the patients were from rural area (65.69%) and belonged to a low socioeconomic and educational status. 30.65% of the patients were housewives, 23.35% drivers, and 16.78% labourers. Predominant mode of transmission was heterosexual contact (94.16%). Most common HIV-related dermatological manifestations were seborrheic dermatitis (74.16%), xerosis (52.5%), generalized skin hyperpigmentation 56 (46.67%), onychomycosis 53 (44.16%), pruritic papular eruption 27 (22.5%), oral candidiasis 21 (17.5%), photo dermatitis 21 (17.5%), and scabies 4 (3.33%). Significant correlation with low CD4+ cell counts was found for oral candidiasis (*P* < 0.0001) and Kaposi's sarcoma (*P* = 0.03), while other disorders such as seborrheic dermatitis (*P* = 0.22), xerosis (*P* = 0.25), and onychomycosis (*P* = 0.08) were not statistically significant.

**Conclusion:**

This study showed high prevalence of dermatological manifestations in HIV-infected subjects, and they occur more frequently with progression of HIV and decline in immune functions. Therefore, early diagnosis and management of skin disorders can improve the quality of life of HIV-infected subjects.

## Introduction

A total of 39.5 million (34.1 million–47.1 million estimated) people were found to be HIV-positive in 2006,[1] this includes the estimated 4.3 million (3.6 million–6.6 million) adults and children who were newly infected with HIV. According to the new, more accurate estimates, the number of people in India living with HIV in 2006 was approximately 2.5 million (2 million–3.1 million) and the adult national HIV prevalence was 0.36%. This confirms that the proportion of people living with HIV/AIDS (PLHA) is lower than previously estimated.[2] The highest number of PLHA is in Andhra Pradesh and Maharashtra. Manipur and Nagaland have the highest prevalence due to small population size. According to the National Aids Control Organization (NACO) 2006, HIV prevalence in Chhattisgarh was 0.17%; it was highest (1.67%) in Manipur and lowest (0.03%) in Himanchal Pradesh.[3] In Chhattisgarh, Durg district has the maximum HIV burden followed by Bastar district. These are categorized as category A and C, respectively.[4]

HIV infection constitutes a major health problem[5–7] worldwide. Dermatological disorders are health problems among HIV positive patients which present with a variety of manifestations.[8,9] Skin diseases cause significant morbidity and may be initial signs of immunosuppression.[10] They affect between 80 and 95% of HIV-infected patients,[11,12] occurring at any time in the course of infection. Skin is often the first and only organ affected during most of the course of HIV disease.[11,13,14] Cutaneous disorders during HIV infection are numerous.[15,16] Some Cutaneous disorders reflect the progression of HIV disease,[13,15] but this relation is still controversial.[16,17] Extensive HIV-related literature has focused on distinctive clinical presentations such as Kaposi's sarcoma, oral hairy leukoplakia, and oral candidiasis.[15,17] However, the findings on careful skin examination of HIV infected patients who present for primary care have received limited attention. Here, we describe systematic dermatological manifestations of HIV infected patients on initial presentation.

## Materials and Methods

This is a prospective study, which was conducted in Govt. Medical College and associated Maharani Hospital, Jagdalpur (Chhattisgarh) between January 2006 and June 2008. Patient's date were collected from OPD, in ward, and from counseling center, and all participants testing positive for HIV at screening were enrolled for ongoing study. There were no specific eligibility criteria. All HIV positive patients who accessed care at hospital were included. Information on demographics, that is, age, sex, height, weight, socioeconomic and educational status, laboratory parameters (CD4+ counts) and treatment regime were noted. A complete medical history and physical examination of patients were done by a dermatologist for optimal evaluation and diagnosis of dermatologic lesions on the basis of clinical appearance. Data were analyzed, using the chi square test for establishing correlation between CD4+ cell counts and various dermatological disorders, *P* value < 0.05 was considered to be clinically significant.

## Results

A total of 137 HIV-infected patients were enrolled in the present study and the following observations were made [Tables 1–4].

**Table 1:** Demographic parameters of HIV infected patients

	No. of cases (%)
Age in years	
Below 19 years	07 (5.1)
20–29	44 (32.11)
30–39	54 (39.41)
40–9	23 (16.78)
50–59	08 (5.83)
>60	01 (0.72)
Grand total	137
Sex wise distribution	
Male	83 (60.58)
Female	54 (39.41)
Grand total	137
Residence	
Urban	47 (34.30)
Rural	90 (65.69)
Grand total	137
Occupations	
Government servants	12 (8.75)
House wives	42 (30.65)
Farmers	07 (5.10)
Business	10 (7.29)
Laborers	23 (16.78)
Drivers	32 (23.35)
Grand total	137
Socioeconomic status	
Upper class	02 (1.45)
Middle class	15 (10.94)
Lower class	109 (79.56)
Below poverty line	11 (8.02)	
Total	137
Religion	
Hindu	131 (95.62)
Muslim	02 (1.45)
Others	04 (2.91)
Total	137
Educational status	
Illiterate	22 (16.05)
Below fifth standard	37 (27)
High school standard	36 (26.27)
Undergraduate	24 (17.51)
Post graduate	18 (13.13)
Total	137
		

**Table 2:** Mode of transmission of HIV infection in patients

	Numbers (%)
Modes of transmission	
Heterosexual	129 (94.16)
Blood transfusion	01 (0.72)
Maternal transmission	07 (5.10)
I.V. drug abuser (syringe)	00 (0)
Homosexual	00 (0)
CD4 count status(at presentation) *n* = 68	
<250/ μl	23 (33.82)
>250/ μl	45 (66.17)
Total	68

**Table 3:** Prevalence of dermatological manifestation in HIV infected patients

Dermatological manifestation	Numbers (%)
Xerosis	63 (52.5)
Pruritic papular eruption	27 (22.5)
Onychomycosis	53 (44.16)
Seborrheic dermatitis	89 (74.16)
Oral candidiasis	21 (17.5)
Icthyosis	03 (2.5)
Erythroderma	06 (5.0)
Diffuse alopecia	08 (6.67)
Tinea unguium	03 (2.5)
Generalized skin hyper-pigmentation	56 (46.67)
Atopic dermatitis	15 (12.5)
Photo dermatitis	21 (17.5)
Psoriasis	00 (0)
Kaposi sarcoma	02 (1.67)
Oral ulcers	21 (17.5)
Scabies	04 (3.33)
Folliculitis	04 (3.33)
Pityriasis versicolor	03 (2.5)
Mycosis (ring worm)	11 (9.16)
Others	03 (2.5)
No skin manifestation	17 (14.16)

**Table 4:** Cutaneous disorders and its correlation with CD4 cell count

Skin manifestation	Range of CD4 counts and no. of cases		*P*-value
		
	<200/ μl (*n* = 23) (%)	>200/ μl (*n* = 45) (%)	
Xerosis	15 (65.2)	23 (51.1)	0.25
Onychomycosis	09 (39.1)	9 (22.2)	0.08
Oral candidiasis	10 (43.4)	02 (04.4)	<0.0001
Seborrheic dermatitis	18 (78.2)	29 (64.4)	0.22
Kaposi sarcoma	02 (08.6)	00 (00)	<0.03

Among 137 HIV patients, 83 (60.58%) were males and 54 (39.41%) were females. Of these, most patients, 54 (39.41%), were in 30–39 years age group (19 females and 35 males), while female prevalence was highest (18.97%) in the age group of 20–29 years. The majority of patients (90, i.e., 65.69%) were residents of rural areas. Of these sufferers, 79.56% belonged to lower socioeconomic status; only 1.45% were from high socioeconomic status. Regarding the occupations of the subjects, the largest group were constituted by house wives (30.65%), followed by drivers 32 (23.35%) and labourers 23 (16.78%). The majority of patients were of poor educational status, 37 (27%) educated only upto fifth standard and 36 (26.27%) upto high school, while 22 (16.05%) were illiterate. A total of 131 (95.62%) subjects were Hindus, the remainder from other religions. The predominant mode of transmission was heterosexual contact (94.16%), only 1 patient (0.72%) was infected through transfusion of infected blood, 7 (5.10%) patients acquired infection via vertical (mother to child) transmission. There was no history of intra venous drug abuse and homosexuality. A total of 68 (49.63%) patients had recent CD4+ cell count, among them 23 (16.78%) had counts less than 250/μl and 45 (32.84%) had more than 250/μl. Most common HIV-related dermatological manifestations were seborrheic dermatitis 89 (74.16%), xerosis 63 (52.5%), generalized skin hyperpigmentation 56 (46.67%), onychomycosis 53 (44.16%), pruritic papular eruption 27 (22.5%), oral candidiasis 21 (17.5%), photo dermatitis 21 (17.5%), and scabies 4 (3.33%). Statistically significant correlation with low CD4+ cell counts was found for oral candidiasis (*P* < 0.0001) and Kaposi's sarcoma (*P* = 0.03); it was insignificant for other disorders such as seborrheic dermatitis (*P* = 0.22), xerosis (*P* = 0.25), and onychomycosis (*P* = 0.08).

## Discussion

The dermatological manifestations in HIV patients, including many opportunistic infections, are very common.[8,9] Results of the present study showed several facts about HIV in the tribal population of Bastar. The majority (90%) of the infected patients belonged to rural areas and belonged to lower socioeconomic strata (79.6%), with income less than Rs. 1500 per month. This indicates poor nutritional status, which itself can accelerate the progression of HIV. A total of 95.62% subjects were Hindus, probably due to high density of tribal Hindu population in this area. The predominant mode of transmission was heterosexual contacts (94.16%); unlike the results of other study,[18] where it has been reported that the modes of transmission was 35.3% homosexual, 27.8% intravenous drug use, and 24.4% heterosexual.

This study was mainly focused on the dermatological manifestations of HIV positive patients attending a tertiary care center (Government Medical College and Maharani Hospital, Jagdalpur) for treatment. We found 87.6% prevalence of dermatological disorders, which is high, similar to the findings of Jeffrey *et al*.[19] (86%) and Pitche *et al*.[20] (82.5%). Goodman *et al*.,[16] reported higher prevalence, whereas a south western France study[18] showed that it is relatively lower (65.3%). Seborrheic dermatitis (74.16%) was the most common dermatological disorder according to our study, which is consistent with other studies[16,21] where seborrheic dermatitis and candidiasis were the most common findings. A study[19] reported dermatophytosis as the most common manifestation. The other frequent dermatologic manifestations in the present study were xerosis (52.5%) and generalized hyperpigmentation of skin (46.67%), the figures being much higher than the previously reported studies[18,19] showing 9.8% and 10.4% and 12%, respectively. Other common manifestation was onychomycosis (44.16%), which was considerably higher than the result of similar study in South Western France (1.8%). The dermatological manifestations increase both in frequency and severity with the progression of HIV and decline in CD4+ cell counts. There was significantly high occurrence of oral candidiasis (*P* < 0.0001), which was similar to many previous studies[18,22,23] and Kaposi sarcoma (*P* < 0.03) among patients with low CD4+ counts (below 200/μl). We used diagnostic technique of clinical evaluation by a dermatologist rather than biopsy or scrapings for dermatological disorders. Although we followed stringent criteria and took all precautions to eliminate human errors, there might have been a possibility in few cases of misdiagnosis.

## Conclusion

We conclude that the main mode of transmission of HIV in the tribal region was heterosexual contact; a critical factor facilitating infections is illiteracy/lack of knowledge and information regarding modes of spread of HIV. The dermatological manifestations have high prevalence among HIV positive subjects; of these, oral candidiasis and Kaposi's sarcoma are significantly correlated with low CD4+ counts. This may provide a clue to the diagnosis of HIV in patients reporting to the health providers, primarily for dermatological manifestations known to be associated with decline in immune function. Thus, patients with such skin complaints may be motivated to report for voluntary counseling and treatment. This also reiterates the need for thorough skin examination in HIV positive patients, and provision of optimal medical care to these patients. Health education and information provided to people in these tribal areas so far has been insufficient and inadequate regarding modes of transmission and methods of prevention of HIV. This statement is especially true for people from lower socioeconomic strata and low educational status, which is an important result emphasized in our study.
